# Evaluation of hygienic conditions of food contact surfaces in a hospital kitchen in Morocco

**Published:** 2019-12

**Authors:** Ghita Benjelloun Touimi, Laila Bennani, Sanae Berrada, Moussa Benboubker, Bahia Bennani

**Affiliations:** 1 Departement of Fundamental Sciences, Laboratory of Human Pathology Biomedecine and environnement, Faculty of Medicine and Pharmacy of Fez (FMPF), Sidi Mohammed Ben Abdellah University (USMBA), Fez, Morocco; 2 Departement of Fundamental Sciences, Faculty of Sciences and Techniques of Fez (FSTF), USMBA, Fez, Morocco; 3 Departement of Fundamental Sciences, The Superior Institute of Nursing Professions and Health Technology of Fez (ISPITS), Fez, Morocco; 4 Department of Nursing, Hassan II University Hospital, Fez, Morocco

**Keywords:** Hospital infection, Food contact surfaces, Hygiene, Food safety, Microbial profile

## Abstract

**Background and Objectives:**

Food in healthcare settings are complementary to medical treatment, hence it should be produced in good sanitary conditions. In fact, hospitalized and immune-compromised patients are more likely to have foodborne infections than the rest of the community. The aim of our study is to evaluate the microbiological quality of food contact surfaces in a hospital kitchen in Morocco.

**Materials and Methods:**

A total of 238 samples was collected from kitchen surfaces and analyzed for total aerobic mesophilic bacteria (AMC), Enterobacteriaceae and *Staphylococcus aureus* count and the presence of *Salmonella* spp., *Pseudomonas* spp. and *Listeria monocytogenes.*

**Results:**

The bacteriological analysis shows that the highest rates of compliance with good hygienic conditions were obtained in baking worktops (77%) and serving meal worktops (50%) and the vegetables cutting boards (45.83%). In contrary, some surfaces show a low level of compliance, such as the raw meat cutting boards (96%). The isolated bacteria were *S. aureus*, coagulase-negative staphylococci, *Escherichia coli*, *Serratia marcescens*, *Serratia odorifera*, *Raoultela ornithiaolytica* and *Pseudomonas aeroguinosa*.

**Conclusion:**

The actual results indicate that the high levels of bacterial counts on kitchen surfaces, presents an evident need to improve the hygienic process and adopt an HACCP system in this facility.

## INTRODUCTION

One of the main hospitals responsibilities is to provide safe and healthy food to the patients (1–3). In fact, several studies reports that a defective practices during food processing leads to food-borne diseases (4, 5). The mortality risk related to nosocomial outbreaks of food-borne infections is considerably higher than outbreaks in community (3).

The contamination during the production process could be related to numerous factors, as unsafe food sources, inadequate cooking, improper holding temperatures, contaminated equipment, unclean work surfaces and poor personal hygiene (6, 7). Some foodborne pathogens have the capacity to form biofilms on food contact surfaces, which represent a potential risk on the meals quality and thereby a real threat to the patient (8, 9). Therefore, an adequate cleaning, effective hygiene process and an evaluation of the presence, spoilage and pathogenic microorganisms are an important elements to assure safety and good quality of food (10, 11). Thus, the microbiological analysis of food contact surfaces allows the identification of indicator bacteria of poor hygienic conditions. Three examples of these indicator bacteria are aerobic mesophilic bacteria (AMC), *Staphylococcus* and Enterobactericeae (12, 13).

Hence, in our knowledge, there is no Moroccan data on the incidence the microbial ecology of food contact surfaces in hospital kitchens. Therefore, the aim of our study is to evaluate the hygiene condition of food contact surfaces in a Moroccan hospital.

## MATERIALS AND METHODS

### Study design and sampling.

Our study was carried out from June 2015 to June 2016 and concerned food contact surfaces samples in a hospital kitchen located in Fez region in Morocco. In this hospital, a private company manages the catering service. It produces more than 1000 meals/day designated to patients and medical staff. The samples were collected once per month and obtained from available materials used in the moment of our sampling (Table 1). Microbiological analysis of the food contact surfaces samples and identifications tests were executed at the Laboratory of Microbiology and Molecular Biology, Faculty of Medicine and Pharmacy of Fez City.

**Table 1.** Food contact surfaces samples examined distribution

**Sample type**	**Number of samples**
Chopping meat devise	26
Knives	10
Weighing machine	16
Sink	20
Recipient	16
Kneading machine	8
Baking worktops	26
Serving meal worktops	26
Raw meat cutting boards	44
Vegetables cutting boards	24
Salads preparation recipients	22
Total	238

The sampling of dry and wet surfaces was done according to Evancho et al. protocol (14). A prepared sterile template was placed on the targeted area ranging from 20 to 100 cm^2^, according to the dimension of the surface to be sampled. The surfaces were swabbed by sterile cotton swabs (Oxoid, UK), pre-moistened into a 5 mL sterile Brain heart Infusion solution (Oxoid, UK) then transported to the laboratory in ice boxes (4°C).

### Bacteriological analysis.

The enumeration and isolation of the total AMC was performed on Plate Count Agar (Biokar Diagnostics, France) after 72 h incubation at 30°C (±1°C). VRBL Agar (Biokar Diagnostics, France) was used to enumerate the Enterobactericeae after 24 h of incubation at 44°C (±1°C). Following incubation, the obtained colonies were streaked into eosin methylene blue agar (EMB) (Biokar Diagnostics, France) in order to discriminate *E. coli* (metallic aspect). Biochemical assays including: Gram straining, Oxidase, Fermentation, Citrate degradation were used to characterizes each isolates. The results were confirmed using API 20E kit (BioMerieux, France). *Pseudomonas* spp. was detected in Cetrimide agar at 37°C± 1°C for 24 h. The detection of staphylococci was done as follows: The swabs were enriched in brain heart infusion broth (Oxoid, Basingstoke, UK) supplemented with 5% sodium chloride at 37°C for 24 h and then plated onto Baird parker (selective media for *Staphylococcus*) and incubated for 24 h at 37°C. The identification was based on microscopic characteristics and biochemical assays including: Gram staining, Catalase, DNase, Mannitol and fermentation. The confirmation of *Staphylococcus aureus* was done with Coagulase test (rabbit plasma). For the detection of *Salmonella* spp., the swabs were incubated for 18–24 h at 37°C (±1°C) then a selective enrichment of 0.1 ml was done in 10 ml of Rappaport-Vassiliadis (RV) (Biokar Diagnostics, France). The RV broth was incubated at 42°C (±1°C) for 18–24 h, then the broth was sub cultured onto Hektoen Agar and incubated at 37°C (±1°C) for 18–24 h. Presumptive positive colonies (non-lactose fermenting with suitable colony morphology) were then confirmed using biochemical tests and API 20E (Biomerieux, France).

*Listeria* isolation and identification were carried out in pre-enrichment Demi-Fraser broth (Biokar Diagnostics, France) 24 h at 30°C. 1 ml of pre-enrichment culture was transferred to 9 ml enrichment Complete Fraser broth, (Biokar Diagnostics, France), and then incubated at 37°C for 24 h. One ml was transferred to PALCAM (Biokar Diagnostics, France), then the plates were incubated at 37°C for 24 to 48 h. Typical colonies considered as possible *Listeria* spp. were purified on Tryptic Soy Agar with 0.6% yeast extract (Oxoid, England) and biochemical tests were used for identification. The suspected isolates of *Listeria monocytogenes* were confirmed using API *Listeria* (Biomerieux, France).

The enumeration results were expressed in log_10_CFU/cm^2^. To interpret our results, we considered the criteria established by Losito et al. (15). This author classified the samples into three categories according to bacteria counts. The samples is considered as: compliant if the bacteria count ranged from 0 to 1.6 log_10_CFU/cm^2^, improvable if the rate is ranged between 1.6 and 2.69 log_10_CFU/cm^2^ and not compliant when it surpassed 2.70 log_10_CFU/cm^2^. These compliance criteria were selected because they were practical, achievable and verifiable for the evaluation of hygiene and sanitation programs of surfaces in food industry and distribution system.

### Statistical analysis.

Descriptive statistics analysis was done using SPSS (Statistical product and services solutions, version 20, SPSS Inc. Chicago, Illinois, USA) software. The means, percentages and averages of the enumerated bacteria and compliance rates were calculated for the analyzed samples.

## RESULTS

### Mean levels of isolated bacteria.

The bacteriological analysis of surfaces samples shows that the mean level of mesophilic bacteria, *S. aureus* and Enterobacteriaceae varies from one sample to another. It ranged between 3.94 log_10_CFU/cm^2^ and 1.56 log_10_CFU/cm^2^ for the mesophilic aerobic bacteria, 1.07 log10CFU/cm^2^ and 3.87 log_10_CFU/cm^2^ for *S. aureus* and for the Enterobacteriaceae the means ranged between 0 and 5.19 log_10_CFU/cm^2^ (Table 2). In fact, the highest count of the aerobic mesophilic bacteria, the Enterobacteriaceae and *S. aureus* was detected in the raw meats worktops with a means of 3.94 log_10_CFU/cm^2^, 1.56 log_10_CFU/cm^2^ and 3.37 log_10_CFU/cm^2^ respectively. Moreover, the lowest levels were noticed in the kneading machines with a mean of 1.56 log_10_CFU/cm^2^ for the AMC, 1.07 log_10_CFU/cm^2^ for the *S. aureus.* Although the Enterobacteriaceae were not detected.

**Table 2.** Minimum and maximum counts of the enumerated bacteria

**Sample types**	**Levels of microorganisms (log_10_ CFU/cm^2^) min-max**		

	**AMC**	***Staphylococcus aureus***	**Enterobactericeae**
Chopping meat devise	1.62–3.54	0.01–6.35	0–4.83
Knives	0.77–5.43	0.01–6.35	0–4.83
Weighing machine	1.86–3.06	0.18–6.22	1.2–3.04
Sink	1.2–4.9	1.56–2.20	1.4–2.6
Recipients	1.57–3.53	3.08–3.54	0–2.93
Kneading machine	0.22–3.34	0–1.07	0
Baking worktops	0.29–4.37	0–2.21	0–2.04
Serving meal worktops	0.62–4.7	0.2–2.89	1.8–2.94
Raw meat cutting boards	3.82–4.62	0–3.87	0–5.26
Vegetables cutting boards	0.66–4	0.5–3.51	1.6–5.10
Salads preparation recipients	0.40–1.60	1.4–2.6	1.4–2.1

AMC: aerobic mesophilic bacteria

The results of enterobacteriaceae identification by API 20E is reported in Table 3. The bacterial identification shows that each sample was contaminated with more than one microorganism. The isolated bacteria were *S. aureus, coagulase-negative staphylococci,* E. coli, *Serratia marcescens*, *Serratia odorifera*, *Raoultela ornithiaolytica* and *P. aeroguinosa* (Table 4).

**Table 3.** results of API 20E of each isolated species

**Identified strains**	**Biochemical profile in API tests**																				

	**ONGP**	**ADH**	**LDC**	**ODC**	**CIT**	**H2S**	**URE**	**TDA**	**IND**	**PV**	**GEL**	**GLU**	**MAN**	**INO**	**SOR**	**RHA**	**SAC**	**MEL**	**AMY**	**ARA**	**OX**
*Serratia marcescens*	−	+	+	+	+	−	+	+	−	+	+	+	+	+	+	+	+	+	−	+	−
*Escherichia coli*	+	−	+	+	−	−	−	−	+	−	−	+	+	−	+	+	+	+	−	+	−
*Serratia odorifera*	+	−	+	+	+	−	−	−	+	+	+	+	+	+	+	+	+	+	+	+	−
*Raoultella ornithinolytica*	+	−	+	+	+	−	+	−	+	−	−	+	+	+	+	+	−	+	−	+	−

ONPG: Ortho-nitrophényl-β-galactoside; ADH: Arginine dihydrolase; LDC: Lysine Decarboxylase; CIT: Citrate; TDA: Tryptophane désaminase; IND: Indole; VP: sodium pyruvate; GEL: Gelatin; GLU: Glucose; MAN: Mannose; INO: inositol; SOR: Sorbitol; RHA: Rhamnose; SAC: Saccharose; MEL: D-melibiose; AMY: amygdalin; ARA: L-arabinose e; OX: Oxidase

**Table 4.** Occurrence of microorganisms in the exanimated surfaces

	**Incidence of Microorganisms in food contact Surfaces; N (%)**								

	***S. aureus***	***SCN***	***E. coli***	***Serratia marcescens***	***Serratia odorifera***	***Raoultela ornithiaolytica***	***P. aeroguinosa***	***Salmonella***	***L.monocytogenes***
Chopping meat devise N= 26	10 (38.46)	5 (19.23)	7 (26.97)	0	3 (11.53)	-	-	-	-
Knives N=10	3 (30)	0	0	0	0	1 (10)	-	-	-
weighing machine N=16	0	4 (25)	10 (62.5)	2 (12.5)	0	0	10 (62.50)	-	-
Sink N=20	7 (35)	20 (100)	15 (75)	5 (25)	0	0	-	-	-
Recipients N=16	3	2 (12.5)	2 (12.5)	3 (18.75)	0	0	-	-	-
kneading machine N=8	1 (12.5)	3 (37.5)	0	0	0	0	-	-	-
Baking worktops N=26	7 (26.92)	12 (46.15)	10 (38.46)	0	0	5 (19.23)	-	-	-
Meal serving worktops N=26	3 (11.53)	10 (38.46)	5 (19.23)	2 (7.69)	0	0	-	-	-
Raw meats cutting boards N=44	20 (45.45)	36 (81.81)	40 (90.90)	9 (20.45)	5 (11.36)	10 (22.72)	30 (68.18)	-	-
Vegetables cutting boards N=24	12 (50)	20 (83.33)	20 (83.33)	6 (25)	2 (8.33)	4 (16.66)	-	-	-
Salads preparation recipients N=22	5 (22.72)	13 (59.09)	8 (36.36)	0	0	0	-	-	-

*S. aureus: Staphylococcus aureus*; SCN: *Staphylococcus* coagulase negative, *E. coli*: *Escherichia coli*; *P. aeroguinosa: Pseu-domonas aeroguinosa*; *L. monocytogenes*: *Listeria monocytogenes*; -: Absent

The incidence of each species is different from one surface to another. The *S. aureus* and coagulase-negative staphylococci were mostly isolated in vegetables cutting boards with a frequency of 50% and 83.31% respectively. In addition, the occurrence of *E. coli* was high in the raw meats worktops with 90.90 %. Furthermore, the highest rate of *P. aeruginosa* was found in the weighing machines (62.50%). Although *Listeria monocytogenes* and *Salmonella* spp. were not detected in any food contact surface.

### Samples compliance according to the selected criteria.

**Fig. 1.**
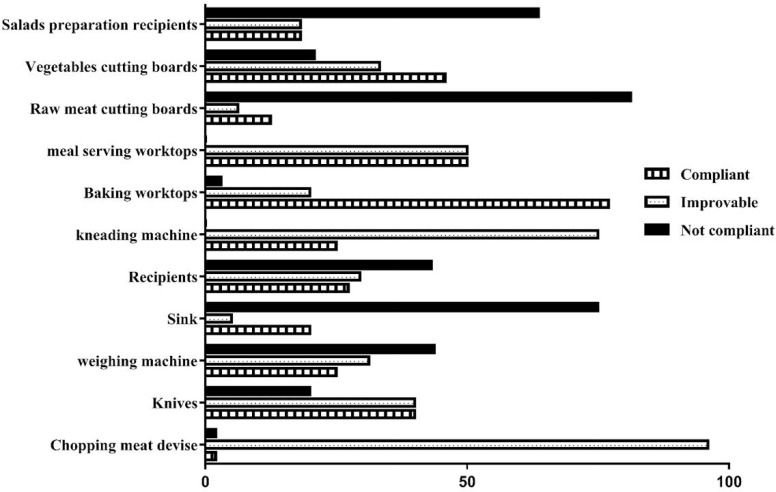
Percentages of compliance according to the selected criteria in different surfaces Compliant: 0 to 1.6 log_10_CFU/cm^2^; Improvable: 1.6 and 2.69 log_10_CFU/cm^2^; Not compliant: 2.70 log_10_CFU/cm^2^.

As noted in Fig. 1, the compliance rates were variable from food contact surface to another. In fact, the highest rates were obtained in baking worktops (77%), the serving meal worktops (50%) and the vegetables cutting boards (45.83%). In contrary, some surfaces shows a low level of compliance with the norms (15), as the: copping meat devise, recipients, the sinks, raw meat cutting boards, weighing machines and the salads preparation recipients (Fig. 1.).

## DISCUSSION

This study aimed to evaluate the microbiological quality of 238 food contact surfaces contact surfaces samples in a Moroccan hospital kitchen in order to provide a new data on the hygienic conditions of food preparation.

AMC counts of the food-processing environment are generally used to estimate the hygiene of the entire food production process (15). High counts of AMC were present on surfaces, equipment, and utensils, indicating unsatisfactory hygienic conditions. Most of the surfaces were inadequate based on Losito et coll. (15) criteria in fact, the means of detected AMC ranged between 3.94 log_10_CFU/cm^2^ and 1.56 log_10_CFU/cm^2^ obtained in Raw meat cutting boards and the kneading machines respectively. These results are comparable to those reported in school kitchen in south Africa (16). However they still higher than those reported in hospital kitchen in Spain (17). These high levels of contamination could be explained by a deficiency of disinfection protocols and cleaning procedures.

Moreover, viable count of Enterobacteriaceae and especially *E. coli* are commonly used to evaluate the hygienic quality of tools and equipment and they are usually known as the most frequent factor causing foodborne diseases and disorders. In this study, the Enterobacteriaceae count was also very high in comparison with several studies as those conducted in Italy (15, 18). The means of these bacteria ranged between zero (in kneading machines) and 5.19 log_10_CFU/cm^2^ (in the raw meats worktops). A high load of *E. coli* generally implies a lack of good manufacturing practices, as well as poor or improper surface sanitization (18).

Furthermore, *S. aureus* is known as an indicator of poor personal hygiene. Food handlers carrying these bacteria are potential source of food contamination during the process of its preparation (19). In this study, the *S. aureus* means level was high than the established criteria. It ranged between 1.07 log_10_CFU/cm^2^ and 3.87 log_10_CFU/cm^2^ detected in kneading machines and raw meats worktops respectively. As *S. aureus* is a major component of the human microbiome, a high degree of handling can enhance its spread to food and food-contact surfaces (13).

According to our results, the raw meats worktops are the most contaminated surfaces and contain the highest counts of bacteria. This can be explained by a cross-contamination from meat and poor hygiene practices. In fact, meat is an ideal media for the development and reproduction of microorganisms, particularly bacteria, so their rapid growth can be expected. With hygiene failing, a cross contamination, from meat to the raw meats worktops and vice versa is inevitable (20). Thus, the high rate of surfaces contamination can constitute a high risk for patients via raw food contamination witch is one of the factors that have been involved in food-borne outbreaks (21, 22).

Moreover, based on the bacteria counts, the microbiological compliance rates were variable from a food contact surface to another. In fact, the highest rates were detected in baking worktops (77%), serving meal worktops worktops (50%) and vegetables cutting boards (45.83%), while the highest rate of non-compliance was found on the raw meats work-tops (81.25%). This last results is similar to those obtained in several studies conducted in Italy (15), Spain (17) and south Africa (16). These higher rates on non-compliance can be explained by the raw nature of the handled materials in those surfaces (raw meats) and the physical nature of the surface. In fact, according to different studies, the contamination risk depends on the surfaces characteristics (smooth, rough, porous, or irregular), their state (new or old equipment) and their handling (left dry or wet after use) (23, 24).

The poor hygiene status of most food surfaces in this study can be attributed to cross-contamination between food materials and food contact surfaces and also to subsequent growth of microorganisms in biofilms (25). In fact, bacteria like *Staphylococcus* and Enterobacteriaceae have a strong ability in forming biofilms which have been known to be highly resistant to antibiotics and to environmental stresses (dry environmental conditions or temperatures) (26). In fact, inadequate cleaning and sanitizing of food contact surfaces, as well as the overall sanitary conditions of food preparation in this facility contribute to the accumulation of food debris and bacteria in biofilms on food contact surfaces. In addition, the lack of proper infrastructure and equipment, incorrect food preparation facilities (27) could participate to the extent of surfaces contamination. Another fact to be considered is the food handlers knowledge on hygiene practices which can be low (9). Thus, defects in hand hygiene may explain the observed levels of contamination. Globally, the non-implementation of the HACCP programs may have negatively influenced the hygienic status of these food contact surfaces and immediate remedial actions are needed.

Overall, food in healthcare settings are considered as complement to medical treatment, therefore it should be produced in good hygienic conditions to prevent food-born nosocomial infections. Consequently, the implementation of strict practices during the production process from primary production to final consumption of hospital meals is obligatory. Thus, more biological hazards may be avoided to the subjects with already compromised health issues.

## CONCLUSION

This study provides the first investigation of the bacteriological quality of food contact surfaces in a Moroccan hospital and reveals areas in need of attention. Based on our actual results, the high levels of bacterial counts on food contact surfaces, presents a strong indication of the need to improve the hygienic process and adopt an HACCP system in this facility, to offer a safe food to the patients.
